# Uncontrolled hypertension in patients with type 2 diabetes: What are the correlates?

**DOI:** 10.1111/jch.14352

**Published:** 2021-08-21

**Authors:** Soghra Rabizadeh, Bahareh Gholami, Shiva Mahmoudzadeh Kani, Armin Rajab, Hossein Farrokhpour, Alireza Esteghamati, Manouchehr Nakhjavani

**Affiliations:** ^1^ Endocrinology and Metabolism Research Center (EMRC) Vali‐Asr Hospital Tehran University of Medical Sciences Tehran Iran

**Keywords:** BMI, hypertension, metabolic syndrome, non‐HDL cholesterol, pulse pressure, type 2 diabetes mellitus

## Abstract

Suboptimal blood pressure (BP) control in patients with type 2 diabetes is associated with adverse micro‐ and macrovascular complications. This study aimed to investigate the predictors of uncontrolled hypertension in an Iranian population with type 2 diabetes. This is a cross‐sectional study of 2612 patients with type 2 diabetes, including 944 patients with hypertension. Controlled and uncontrolled hypertension were assessed. Multivariate logistic regression modeling was used to determined independent predictors of uncontrolled hypertension. Of 2612 patients with type 2 diabetes, 944 (36.1%) patients had hypertension. Of all patients with hypertension, 580 (61.4%) were still on monotherapy. Uncontrolled hypertension was detected in 536 participants (56.8%). Patients with uncontrolled hypertension had significantly higher body mass index (BMI) (29.8±4.8 vs. 28.6±4.6), waist circumference (99.11±10.95 vs. 96.68±10.92), pulse pressure (67.3±17.3 vs. 48.4±10.7), total cholesterol (177.1±45.5 vs. 164.3±40.5), non‐HDL cholesterol (133.0±43.5 vs. 120.1±38.7), triglycerides (175.7±80.3 vs. 157.4±76.7), and Atherogenic Index of Plasma (AIP) (0.57±0.23 vs. 0.52±0.24) (*p <* *.05 for all of them*) compared to patients with controlled hypertension. Multivariate logistic regression analysis revealed that uncontrolled hypertension was significantly associated with BMI (*p *= .001), pulse pressure (*p *= .001), total cholesterol (*p *= .006), and non‐HDL cholesterol (*p *= .009). In patients with triglycerides levels > 200 mg/dl non‐HDL cholesterol had a significant correlation with uncontrolled hypertension (OR = 4.635, CI95%:1.781–12.064, *p* = .002). In conclusion, BMI, pulse pressure, total cholesterol, and non‐HDL cholesterol are significant predictors of uncontrolled hypertension in patients with type 2 diabetes. Also, ineffective monotherapy, medical inertia and patients’ non‐compliance were other contributors to the uncontrolled hypertension.

## 1 INTRODUCTION

International Diabetes Federation (IDF) statistics of 2019 showed that the global prevalence of diabetes between the ages of 20 and 79 years is 9.3%. There were 54.8 million people with diabetes in the middle east and north Africa region in 2019, and this number is expected to rise to 107.6 million in 2045.^1^

The prevalence of hypertension among patients with type 2 diabetes is relatively high. This elevated blood pressure (BP) exacerbates both micro‐and macrovascular complications of diabetes mellitus, including retinopathy, nephropathy, coronary artery disease, an impact illustrated particularly in the ADVANCED trial.^2^, ^3^, ^4^, ^5^, ^6^ It is well documented that reducing BP when systolic BP values are higher than140 mm Hg is associated with a reduction in cardiovascular mortality among diabetic patients with hypertension.^7^ As demonstrated by the hypertension optimal treatment study (HOT) trial and systolic hypertension in Europe (Syst‐Eur) study, patients with diabetes and hypertension experience lower rates of adverse cardiovascular events after BP control compared with patients without diabetes.^8^, ^9^ According to the United Kingdom prospective observational study, in patients with diabetes, every 10 mm Hg reduction in mean systolic BP decreased diabetic‐related deaths by 15%, myocardial infarction by 11%, and microvascular complications by 13%.^10^ However, even with the apparent benefits regarding BP control, a recent report of the national program for prevention and control of diabetes in Iran showed that 56.7% of patients with concurrent hypertension and diabetes had uncontrolled BP.^11^

Various studies had conducted to determine the main predictors of uncontrolled hypertension to help better strategize national health policies.^12^, ^13^, ^14^ However, determinants of uncontrolled hypertension among patients with *diabetes* need to be further studied. Given the importance of this matter, we aimed to investigate predictors of uncontrolled hypertension among patients with diabetes attending the diabetes clinic of Vali‐Asr Hospital.

## 2 MATERIALS AND METHODS

### 2.1 Study design and patients

This was a cross‐sectional study using the data extracted from an ongoing prospective cohort study in patients with type 2 diabetes mellitus who were visited in the diabetes clinic of Vali‐Asr Hospital affiliated with Tehran University of Medical Sciences. Patients with type 2 diabetes were included in the study according to the criteria of the American Diabetes Association (ADA, 2019). The exclusion criteria for this study were type 1 diabetes mellitus, age < 30, and a history of cancer. Patient's medical records were assessed to identify hypertensive patients (Hypertension was defined according to 2019 ADA guideline with a systolic BP of ≥140 mm Hg or diastolic BP of ≥90 mm Hg or use of antihypertensive medication). Measurement of systolic and diastolic BP was performed in the seated position and after 10 min of resting with a standard digital sphygmomanometer. Furthermore, an adapted to the size BP cuff was used in patients with BMI > 30. The measurement of BP was repeated after 15 min, and the average was reported. In the current study, uncontrolled hypertension was defined as having systolic BP > 140 or diastolic BP > 90 in hypertensive patients in the first visit of this study. Briefly, we had three groups in this study: 1‐Normotensive (without hypertension history), 2‐Controlled hypertensive 3‐Uncontrolled hypertensive individuals. A total of 2612 patients with diabetes were enrolled in the current study. The study protocol was approved by the ethics committee of the Tehran University of Medical Sciences.

### 2.2 Data collection

Patient characteristics such as age, sex, height, weight, waist circumferences, BP, smoking, duration of diabetes and hypertension, medication and laboratory measurements including fasting blood sugar (FBS), hemoglobin A1c(HbA1c), total cholesterol (TC), low‐density lipoprotein cholesterol (LDL‐C), high‐density lipoprotein cholesterol (HDL‐C), triglyceride (TG), and serum creatinine were extracted from medical records of the first visit. Non‐HDL cholesterol is measured by subtracting HDL‐C from total cholesterol. Body mass index (BMI) (kg/m²) was calculated by weight (kg) divided by square height (m²). The estimated GFR was calculated using the Cockcroft‐Gault formula in each participant. Albuminuria was defined as having 24 h urine albumin of more than 30 mg. In the current study, NCEP ATP III criteria were used to determine metabolic syndrome.^15^ Positive coronary artery disease included all the previous cardiac events which led to having unstable angina, CCU (critical care unit) admission, PCI (percutaneous coronary intervention), or CABG (coronary artery bypass graft). BMI cut‐off point of 30 was used for obesity. The atherogenic index of plasma (AIP) was calculated as Logaritm10 (TG/HDL‐C).

### 2.3 Statistical analysis

We used statistical software IBM SPSS version 25 for statistical analysis. Data distribution was explored, and normality tests were performed. For continuous variables, data are presented as mean ± SD or median)Interquartile range) and for categorical variables, it is presented as numbers (percentages). Statistical significance was defined as a two‐tailed *p*‐value of less than .05. A two‐sample t‐test was used for comparison between two independent groups with a normal distribution. ANOVA with post hoc tests was performed for comparison between three normally distributed independent groups. For non‐normally‐distributed variables, we used Mann‐Whitney U and Kruskal Wallis for comparison between two and three groups, respectively. Also, the chi‐square test was applied for categorical variables. The study analysis for calculating uncontrolled hypertension predictors is limited to the subset of diabetic patients with hypertension. We categorized the data based on good treatment target cut‐offs. We then used univariate binary logistic regression to identify significant determinants of BP control based on these categorized data. We fitted these determinants into a binary logistic model with BP control as a dependent variable to determine significant predictors of BP control, the variables with unadjusted *p*‐value < .2 for uncontrolled hypertension were recruited in the model. Collinearity between variables was tested by the correlation matrix. Non‐HDL cholesterol and TC, also, TG and AIP had a significant correlation with each other (*r *= 0.73, *p *= .001, and r = 0.75, *p *< .001, respectively). To test both of their association with the outcome variable, we used them in two separate logistic regression models with other variables included.

## 3 RESULTS

After excluding 353 patients, our study population included 2259 patients with type 2 diabetes. We identified 944 individuals with hypertension history, and from these patients, 536 (56.8%) had uncontrolled hypertension. The demographic and laboratory characteristics of all of our study participants with type 2 diabetes are illustrated in Table 1. The mean age of our study participants was 59.2±9.8 years, and 65.4% of them were women. There was a significant difference between controlled and uncontrolled hypertension groups regarding BMI, waist circumference, Pulse pressure, TG, TC, non‐HDL cholesterol, and FBS values(*p *< .05). But no significant difference was observed between these two hypertension groups considering age, sex, smoking, duration of diabetes, duration of hypertension, HDL‐C, LDL‐C, HbA1c, e‐GFR, albuminuria, CAD, metabolic syndrome, medications including anti‐diabetic, antihypertensive, and lipid‐lowering agents (*p *> .05). About 87.2% of individuals with controlled hypertension and 90.8% with uncontrolled hypertension had metabolic syndrome (*p *= .13).

**TABLE 1:** Characteristics of patients with type 2 diabetes regarding hypertension status

Characteristic	Uncontrolled hypertension (*N* = 536/56.8%)	Controlled hypertension (*N* = 408/43.2%)	Total HTN present (*N* = 944)	Normotensive (*N* = 1315)	*p*‐value
Age (years)	59.2±10.0	59.2±9.6	59.2±9.8	53.4±11.1^#^	<.001
Male sex (*n*(%))	193 (36.0%)	133 (32.6%)	326 (34.5)	575 (43.7%)^#^	<.001
Waist circumference (cm)					
Women	99.4±11.4*	97.1±11.7	98.4±11.6	93.9±12.3^#^	<.001
Men	98.6±10.0*	95.8±8.9	97.5±9.6	94.1±10.9^#^	<.001
BMI (kg/m^2^)	29.8±4.8*	28.6±4.6	29.3±4.7	27.67±4.8^#^	<.001
Overweight **	232 (43.9%)*	166 (41.4%)	398 (42.8%)	603 (45.8%)^#^	<.001
Obese ***	224 (42.4%)*	145 (36.2%)	369 (39.7%)	427 (32.4%)^#^	<.001
Smoker—yes (%)	55 (10.3%)	42 (10.3%)	97 (10.3%)	159 (12.1%)	.400
DM duration (year) [median(interquartile range)]	8(3‐14)	7(3‐15)	8(3‐15)	5(2‐6.5)	<.001
HTN duration (year) [median(interquartile range)]	4(2‐10)	4(2‐9)	4(2‐9)		.230
CAD —yes	135 (25.2%)	104 (25.5%)	239 (25.3%)	155 (11.8%)^#^	<.001
Pulse pressure (mm Hg)	67.3±17.3*	48.4±10.7	59.1±17.5	48.5±13.7^#^	<.001
Systolic BP (mm Hg)	154.1±15.5	121.0±11.7	131.2±20.9	125.2±18.2	<.001
Diastolic BP (mm Hg)	86.8±11.1	72.6±9.2	78.4±11.8	76.8±11.0	<.001
FBS (mg/dl)	174.8±63.5	169.0±61.1	172.5±62.5	190.1±80.1^#^	<.001
HbA1c (%) (mmol/mol)	8.0±1.7 64.38±18.85	7.9±1.8 63.59±19.74	8±1.7 64.10±19.25	8.35±1.9^#^ 67.84±21.75	<.001
Total Cholesterol (mg/dl)	177.1±45.5*	164.3±40.5	171.5±43.8	182.28±46.6	<.001
LDL‐C					
CAD :yes	96.6±33.2	85.9±27.7	91.3±31.5	96.9±46.1	.053
CAD: no	92.8±34.9	88.5±31.0	90.9±33.3	102.2±36.8^#^	.002
HDL ‐C					
Women	44.9±9.6	44.9±9.9	44.9±9.7	47±12.5^#^	.010
Men	41±9.7	40.0±9.1	40.5±9.5	41.68±11.6	.350
non‐HDL‐C	133.0±43.5*	120.1±38.7	127.3±41.9	136.9±45.3^#^	<.001
Triglycerides (mg/dl)	175.7±80.3*	157.4±76.7	167.9±79.2	178.5±131.5^#^	.012
AIP	0.57±0.23*	0.52±0.24	0.55±0.24	0.54±0.29	.011
Albuminuria—yes	32 (6.0%)	22 (5.4%)	54 (5.7%)	56 (4.2%)	.230
e‐GFR—cc/min	84.8±31.2	85.3±29.1	85.0±30.3	97.38±33.1^#^	.010
MetS —yes (*n*(%))	488 (91.0%)	357 (87.5%)	845 (89.5)	685 (52.1%)^#^	<.001
Antiglycemic Treatment (*n*(%))					.210
Diet	35 (6.5%)	32 (7.8%)	67 (7.1%)	138 (10.5%)	
OAD	348 (64.9%)	256 (62.7%)	604 (64.0%)	873 (66.4%)	
Insulin	63 (11.7%)	54 (13.2%)	117 (12.4%)	164 (12.5%)	
OAD+ insulin	90 (16.8%)	66 (16.2%)	156 (16.5%)	140 (10.6%)	
Statin use—yes n(%)	524 (97.8%)	397 (97.3%)	921 (97.6%)	1270 (96.6%)	.602
Hypertension drug (*n*(%))					.609
Nothing	181 (33.8%)	127 (31.1%)	308 (32.6%)		
Monotherapy	327 (61.0%)	253 (62.0%)	580 (61.4%)		
Combined	28 (5.2%)	28 (6.9%)	56 (5.9%)		

Data are presented as mean ± SD or median (Interquartile range) for continuous variables and n(%) for categorical variables as appropriate. BP < 140/90 was regarded as controlled blood pressure.

*Abbreviations*: HTN, Hypertension; BMI, Body mass index; DM, Diabetes mellitus; CAD, Coronary artery disease; BP, Blood pressure; FBS, Fasting blood sugar; LDL‐C, Low density lipoprotein cholesterol; HDL‐C, High density lipoprotein cholesterol; non‐HDL‐C, Non high‐density lipoprotein; MetS, Metabolic syndrome; e‐GFR, Estimated glomerular filtration rate; OAD, Oral anti‐diabetic drugs; AIP, Atherogenic index of plasma.

* Significant difference between controlled and uncontrolled hypertension groups.

^#^ Significant difference between normotensive and hypertensive groups.

** Overweight: 25 kg/m^2^≤BMI < 30 kg/m^2^.

*** Obesity: 30≤BMI kg/m^2^.

BMI was significantly higher in the uncontrolled hypertension group compared to the controlled hypertension group (29.8±4.8 vs. 28.6±4.6 kg/m^2^, *p* < .001). Obesity was significantly higher in patients with uncontrolled hypertension than patients with controlled hypertension (42.4% vs. 36.2%, *p* < .001). Additionally, both controlled and uncontrolled hypertension groups had significantly higher BMI than normotensive individuals. (28.6±4.6 vs. 27.67±4.8 kg/m^2^, *p* value = .001; 29.8±4.8 vs. 27.67±4.8 kg/m^2^, *p* < .001 respectively)

Pulse pressure was significantly higher in uncontrolled hypertension compared to the controlled hypertension group (67.3±17.3 vs. 48.4±10.7 mm Hg, *p* < .001. Also, pulse pressure in uncontrolled hypertensive patients was higher than their normotensive peers (67.3±17.3 vs. 48.51±13.7; *p* < .001). It should be noted that the age difference between patients with hypertension and patients without hypertension have a potential role in the pulse pressure variation.

There was a statistically significant difference between controlled and uncontrolled hypertension groups regarding TG (157.4±76.7 vs. 175.7±80.3 mg/dl; *p* < .001) and TC (164.3±40.5 vs. 177.1±45.5 mg/dl, *p *< .001). Additionally, we observed significant difference between controlled hypertensive patients and their normotensive peers with regard to TG (157.4±76.7 mg/dl vs. 178.52±131.5 mg/dl, *p* = .001) and TC (164.3±40.5 mg/dl vs. 182.28±46.6 mg/dl, *p* < .001).Furthermore, patients with uncontrolled hypertension had significantly higher non‐HDL cholesterol compared to patients with controlled hypertension (133.09±43.5 mg/dl vs. 120.1±38.7 mg/dl, *p *< .001). Also, AIP was significantly higher among patients with uncontrolled hypertension in comparison with controlled ones (0.57±0.23 mg/dl vs. 0.52±0.24 mg/dl, *p *= .005).

HbA1c was not significantly different between controlled and uncontrolled hypertension groups (7.9±1.8 % vs. 8.0±1.7 %, *p* = .57). However, it was significantly higher in normotensive patients than uncontrolled hypertensive patients. (8.35±1.9 % vs. 8.0±1.7%, *p* = .004). Similarly, HbA1C was higher in normotensive patients compared to controlled hypertensive group (8.35 ±1.9 % vs. 7.9±1.8 %, *p *= .002) .FBS was significantly different between normotensive (190.1±80.1 mg/dl) and controlled (169.0±61.1 mg/dl) or uncontrolled hypertensive patients (174.8 ±63.5 mg/dl) (*p *= .001).

The unadjusted cross‐tabulation analysis showed significant differences only in the distribution of categorized BMI, total cholesterol, triglyceride, non‐HDL‐C, and AIP. AIP was categorized by its median (Table 2).

**TABLE 2:** Significant results of univariate analysis for comparison between controlled and uncontrolled hypertension groups after categorizing the data

Variable	Total (HTN present) (*N* = 944)	Controlled hypertension (*N* = 408)	Uncontrolled hypertension (*N* = 536)	*p* value
BMI (*n* (%))				.002
<25	164 (17.4%)	91 (22.3%)	73 (13.6%)	
25–30	404 (42.8%)	169 (41.4%)	235 (43.8%)	
>30	376 (39.8%)	148 (36.3%)	228 (42.5%)	
Total cholesterol				.001
<200	736 (78.0%)	343 (84.1%)	393 (73.3%)	
200–240	143 (15.1%)	48 (11.8%)	95 (17.7%)	
>240	65 (6.9%)	17 (4.2%)	48 (9.0%)	
Triglyceride				.006
<150	483 (51.2%)	236 (57.8%)	247 (46.1%)	
150–200	203 (21.5%)	73 (17.9%)	130 (24.2%)	
>200	258 (27.3%)	99 (24.3)	159 (29.7%)	
Non‐HDL‐C				<.001
<130	539 (57.1%)	264 (64.7%)	275 (51.3%)	
130–160	223 (23.6%)	89 (21.8%)	134 (25.0%)	
>160	182 (19.3%)	55 (13.5%)	127 (23.7%)	
AIP				.009
= < 0.54	460 (48.7%)	222 (54.4%)	238 (44.4%)	
>0.54	484 (51.3%)	186 (45.6%)	298 (55.6%)	

Cross tabulation results are shown. Note: Only significant comparisons are shown.

*Abbreviations*: HTN, Hypertension; BMI, Body mass index; non‐HDL‐C, Non high‐density lipoprotein cholesterol; AIP, Atherogenic index of plasma.

Logistic regression models after adjusting for other variables were shown in Table 3. Models revealed that higher BMI, higher pulse pressure, higher cholesterol and, higher non‐HDL‐C were more prevalent among patients with uncontrolled hypertension (*p *< .05), while Triglyceride and AIP did not have any significant difference between groups after adjustment for other variables. Furthermore, models with non‐HDL‐C had greater R square than models with total cholesterol (R^2 ^= 0.431 in model 2 and R^2 ^= 0.430 in model 4 Vs. R^2 ^= 0.420 in model 1 and R^2 ^= 0.424), it seems that higher levels of non‐HDL‐C are more suggestive of uncontrolled hypertension compared to total cholesterol. Moreover, based on model 2, the odds ratio of uncontrolled hypertension in patients with non‐HDL‐C more than 160 mg/dl was 2.391 compared to patients with non‐HDL‐C lower than 130 mg/dl (*p *= .002).

**TABLE 3:** Multivariate logistic regression analysis showing adjusted Odds ratio (OR) for uncontrolled hypertension

	Model 1(R^2^ = 0.420)		Model 2 (R^2^ = 0.431)		Model 3 (R^2^ = 0.424)		Model 4 (R^2^ = 0.430)	
	Adjusted OR (95% CI)	*p* value	Adjusted OR (95% CI)	*p* value	Adjusted OR (95% CI)	*p* value	Adjusted OR (95% CI)	*p* value
BMI (Ref: BMI < 25)		.**001**		.**004**		.**001**		.**001**
25‐30	**2.006** (1.203‐3.345)	.**008**	**1.812** (1.068‐3.074)	.**028**	**1.970** (1.148‐3.383)	.**014**	**1.775** (1.037‐3.036)	.**036**
>30	**2.663** (1.576‐4.498)	**<.001**	**2.535** (1.471‐4.367)	.**001**	**2.794** (1.611‐4.845)	**<.001**	**2.546** (1.471‐4.406)	.**001**
Pulse pressure (1 mm Hg increment)	**1.096** (1.080‐1.112)	**<.001**	**1.099** (1.082‐1.116)	**<.001**	**1.098** (1.081‐1.115)	**<.001**	**1.099** (1.082‐1.116)	**<.001**
Triglyceride (Ref: triglyceride < 150)		.226			–	–	–	–
150‐200	1.470 (0.917‐2.355)	.109	1.423 (0.869‐2.330)	.161	–	–	–	–
>200	1.294 (0.826‐2.025)	.260	1.131 (0.692‐1.849)	.624	–	–	–	–
AIP (Ref: AIP < 0.54)	–	–	–	–	1.313 (0.897‐1.923)	.162	1.470 (0.917‐2.355)	.518
Total Cholesterol (Ref: Total Cholesterol < 200)		.**008**	–	–		.**006**	–	–
200‐240	**1.956** (1.147‐3.337)	.**014**	–	–	**2.049** (1.186‐3.537)	.**010**	–	–
>240	**2.549** (1.107‐5.872)	.**028**	–	–	**2.658** (1.124‐6.284)	.**026**	–	–
non‐HDL‐C (Ref: non‐HDL‐C < 130)	–	–		.**001**	**–**	**–**		.**001**
130‐160	–	–	**1.868** (1.178‐2.962)	.**008**	**–**	**–**	**1.914** (1.208‐3.031)	.**006**
>160	–	–	**2.391** (1.382‐4.137)	.**002**	**–**	**–**	**2.509** (1.463‐4.302)	.**001**

Multivariate logistic regression analysis was used to calculate significant predictors of uncontrolled hypertension after adjusting for other variables.

*Abbreviations*: BMI, Body mass index; Ref, Reference group; AIP, Atherogenic index of plasma; non‐HDL‐C, Non high density lipoprotein cholesterol; 95%CI, 95% confidence interval; OR, Odds ratio.

**FIGURE 1:**
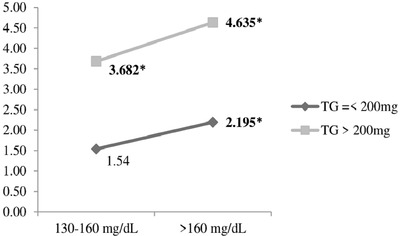
The comparison of odds ratios of non‐HDL‐C level and blood control status based on serum triglyceride levels. Reference group: non‐HDL‐C = < 130 mg/Dl. *Significant difference with reference group (*p* < .05). TG: Triglyceride; non‐HDL‐C: none high‐density lipoprotein cholesterol

In Table 4, patients were stratified based on serum triglyceride levels lower and higher than 200 mg/dl. Binary logistic regression showed that higher levels of non‐HDL‐C had a stronger correlation with uncontrolled hypertension among patients with serum triglyceride levels of more than 200 mg/dl (OR = 4.635, CI95%:1.781‐12.064, *p *= .002) (Figure 1). Also, in patients with serum triglyceride levels higher than 200 mg/dl, the correlation between BMI and uncontrolled hypertension did not remain significant (*p *= .798).

**TABLE 4:** Binary logistic regression analysis showing adjusted Odds ratio (OR) for uncontrolled hypertension based on serum triglyceride level

	Triglyceride = < 200 mg (R^2^ = 0.444)		Triglyceride > 200 mg (R^2^ = 0.422)	
	Adjusted OR (95% CI)	*p* value	Adjusted OR (95% CI)	*p* value
BMI (Ref: BMI < 25)		.**010**		.798
25‐30	**2.053** (1.079‐3.907)	.**028**	1.109 (0.330‐3.730)	.867
>30	**3.440** (1.556‐7.605)	.**002**	1.456 (0.382‐5.549)	.582
Pulse pressure (1 mm Hg increment)	**1.107** (1.086‐1.127)	**<.001**	**1.090** (1.059‐1.122)	**<.001**
non‐HDL‐C (Ref: non‐HDL‐C < 130)		.**049**		.**003**
130‐160	1.540 (0.884‐2.681)	.127	**3.682** (1.433‐9.458)	.**007**
>160	**2.195** (1.079‐4.463)	.**030**	**4.635** (1.781‐12.064)	.**002**

Binary logistic regression analysis was used to calculate significant predictors of uncontrolled hypertension after adjusting for other variables.

*Abbreviations*: BMI, Body mass index, Ref, Reference group, non‐HDL‐C, Non high density lipoprotein cholesterol; 95%CI, 95% confidence interval; OR, odds ratio.

## 4 DISCUSSION

This study highlighted four important characteristics associated with uncontrolled hypertension among patients with diabetes, including BMI, pulse pressure, total cholesterol, and non‐HDL cholesterol.

### 4.1 BMI

This study pointed out that BMI is a significant predictor for uncontrolled BP status in patients with type 2 diabetes, similar to previous studies.^16^, ^17^, ^18^ In this study, uncontrolled hypertension was significantly higher in both patients with overweight and obesity. In comparison to patients with BMI of less than 25, the odds of uncontrolled hypertension increased by 2.25 times in patients with BMI of 25–30 (*p *= .004) and by 2.87 times in patients with BMI of more than 30 (*p *< .001). Likewise, both controlled and uncontrolled hypertensive patients had higher BMI than patients without hypertension. We noted a relatively high frequency of obesity among individuals with controlled and uncontrolled hypertension (36.2% and 42.4%, respectively). Obesity contributes to hypertension by several mechanisms, including sympathetic nervous system activation,^19^ increased free fatty acids,^20^ renin‐angiotensin system activation,^21^ and angiotensin II production in adipose tissue.^22^ Hyperinsulinemia, a connecting factor between obesity, diabetes, and metabolic syndrome, is also associated with hypertension through anti natriuretic, sympathomimetic effects, and RAS activation.^23^ Furthermore, obesity has been considered a major risk factor for Obstructive sleep apnea (OSA). A strong body of evidence has shown the bidirectional association between OSA and hypertension.^24^ Although the number of patients with OSA is not provided in this study, given the strong relationship between obesity and OSA and the higher rate of obesity in this cohort, it can be estimated that the higher rate of uncontrolled hypertension is partly attributed to the OSA in the patients with higher BMI values.

### 4.2 Metabolic syndrome

Metabolic syndrome is a major cause of cardiovascular mortality.^25^ In this study prevalence of metabolic syndrome for patients with controlled and uncontrolled hypertension was 87.2% and 90.8%, respectively. These numbers are higher than the national prevalence of metabolic syndrome, which is 32.9% for the Iranian population.^26^ Walter Zidek and colleagues showed that in patients with hypertension, BP control which was not achieved by antihypertensive therapy, was associated with metabolic syndrome and its components. Nevertheless, we didn't see any association between uncontrolled hypertension and metabolic syndrome. This might partly be because all of our patients already had two of the metabolic components including diabetes, and hypertension. However, the former study population was not limited to diabetic patients.^27^

### 4.3 Pulse pressure

Pulse pressure which is the difference between systolic and diastolic BP is known to be a significant independent risk factor for cardiovascular mortality.^28^ Blacher J and colleagues calculated an increased risk of coronary disease by 13% and cardiovascular mortality by 20% with every 10 mm Hg increase in pulse pressure.^29^ We observed a positive association of uncontrolled hypertension with wide pulse pressure. Higher pulse pressure associated with uncontrolled hypertension in diabetes amplifies cardiovascular events in this subset of patients. Targeting pulse pressure and arterial stiffness would be a smart approach for minimizing cardiovascular mortality.

### 4.4 Lipids

We observed that higher triglycerides, total cholesterol, and non‐HD levels were associated with uncontrolled hypertension in univariate analysis. However, after adjusting for other variables in the final regression model, only total cholesterol and non‐HDL cholesterol remained significant predictors for uncontrolled BP. Comparably in a prospective cohort, Ruben O and colleagues showed that the highest quintile of TC and non‐HDL cholesterol increased the likelihood of developing hypertension by 23%, 39%, respectively.^30^ According to the Wen and colleagues study, dyslipidemia, particularly when demonstrated as lipoprotein ratios, is a predictor of arterial stiffness.^31^ Arterial stiffness, measured by pulse wave velocity, has been linked to hypertension through various mechanisms contributing to uncontrolled hypertension.^32^ Normotensive patients had higher TG and TC than controlled hypertensive patients. This might be explained by the different drug adherence in these patients. Moreover, the models with non‐HDL‐C were more suggestive of uncontrolled hypertension compared to models with total cholesterol. It may be due to the fact that the calculation of non‐HDL‐C is related to both total cholesterol and HDL‐C levels. The atherogenic index of plasma and triglyceride did not remain in models; however, patients with uncontrolled hypertension had significantly higher levels of AIP and triglyceride.

The current study showed that higher levels of non‐HDL‐C had a stronger correlation with uncontrolled hypertension among patients with serum triglyceride levels of more than 200 mg/dl (OR = 4.635, *p* = .002) (Figure 1). Besides, in patients with serum triglyceride levels higher than 200 mg/dl, the correlation between BMI and uncontrolled hypertension did not remain significant (*p* = .798).

Non‐HDL cholesterol is the sum of LDL and VLDL. It includes all Apo B containing lipoproteins and represents a surrogate marker for total Apo B. Apolipoprotein B is a major atherogenic lipoprotein and a strong predictor for the severity of coronary heart disease. When triglyceride levels are lower than < 200 mg/dl, non‐HDL‐C correlates highly with LDL‐C and non‐HDL provides little additional power to predict coronary heart diseases. However, when triglycerides levels are more than 200 mg/dl, VLDL cholesterol levels are elevated, and LDL‐C levels correlate less with non‐HDL‐C. Consequently, in individuals with high triglycerides concentrations, non‐HDL‐C is a better representative of atherogenic lipoproteins than LDL‐C. In very high triglycerides levels (eg, ≥500 mg/dl), non‐ HDL‐C levels have a less predictive value for coronary heart disease.^33^, ^34^, ^35^, ^36^

### 4.5 Anti‐hyperglycemic drugs

Existing literature has not reached a consensus on the effect of anti‐diabetic drugs on BP. Our results showed no significant difference between controlled and uncontrolled hypertensive and normotensive groups regarding the type of anti‐diabetic medication, including oral anti‐diabetic agents, insulin, or both, and the type of oral anti‐diabetic agents themselves. Katsi V and colleagues reported that thiazolidinediones, DPP‐4 inhibitors, and SGLT2 inhibitors could have a BP‐lowering effect, and sulfonylureas might increase BP.^37^ In a recent study Packer and colleagues demonstrated that receiving empagliflozin (SGLT2 inhibitor) in patients with heart failure was associated with BP control, renal protection, and lower risk of hospitalization for heart failure or cardiovascular death, both in patients with diabetes mellitus and patients without diabetes mellitus.^38^ Alemi and colleagues in a prospective cohort study, found no significant change regarding BP in normotensive patients with type 2 diabetes receiving metformin, glibenclamide + metformin, metformin + insulin or insulin therapy.^39^ This finding was consistent with the current study results.

Like previous studies, no sex disparity was seen considering BP control in patients with diabetes^18^, ^40^, ^41^

### 4.6 Improvement of blood pressure control

Current evidence supports starting antihypertensive therapy with a two‐drug combination as the first line, based on the latest European society of cardiology and the European society of hypertension(ESC/ESH) 2018.^42^ There are several reasons behind this recommendation, particularly medical inertia defined as failure of health care providers to intensify treatment when indicated^43^ and patients’ non‐compliance to the therapy, a factor with much more importance than previously anticipated.^44^ In this cohort, not only patients with hypertension received insufficient treatment despite inadequate control of BP levels, but also patients on oral anti diabetic monotherapy had markedly elevated levels of HbA1c, reflecting high rates of medical inertia or individuals’ non‐compliance. This is in line with previous data showing endorsement of ineffective monotherapy or suboptimal doses in spite of undesirable results in many patients^45^ in addition to low adherence to the treatment.^44^ Interestingly, it has been demonstrated that 60% of all patients candidate for dual therapy, are still under monotherapy.^45^ This is in line with the 61.4% monotherapy rate among all patients with hypertension, reported in this study.

### 4.7 Limitations

The cross‐sectional nature of our study makes it hard to conclude the causality between the predictors and the outcome variable. Also, the number of patients with OSA was not available.

## 5 CONCLUSIONS

Although most hypertensive patients were on antihypertensive medication, 56.8% of them had uncontrolled BP. This study suggested that uncontrolled hypertension in patients with type 2 diabetes was positively associated with high BMI, pulse pressure, total cholesterol, and non‐HDL cholesterol. This study showed that non‐HDL cholesterol is a strong correlate of uncontrolled hypertension in patients with both type 2 diabetes and hypertriglyceridemia. Additionally, it was demonstrated that despite suggesting dual antihypertensive therapy as the first line by the latest evidence, more than 50% of patients with hypertension were still on monotherapy. Furthermore, the role of ineffective monotherapy, medical inertia, and patients' non‐compliance in uncontrolled hypertension was illustrated. Identifying these predictors could be of great importance in that, it contributes to better strategic planning for tackling hypertension problems among high‐risk patients.

This research is not supported by any particular person or organization

## CONFLICT OF INTEREST

The authors declare that they have no conflict of interest.

## AUTHOR CONTRIBUTIONS

*Soghra Rabizadeh*: conceptualization, methodology, investigation, writing ‐ Original Draft, data curation, writing ‐ Original Draft visualization, critically revising the manuscript. *Bahareh Gholami*: investigation, writing ‐ Original Draft.: data curation, methodology, writing ‐ Original Draft, visualization, critically revising the manuscript. Shiva Mahmoudzadeh Kani: methodology, investigation, writing ‐ Original Draft, methodology, writing ‐ Original Draft, visualization, review the revised version. *Armin Rajab*: validation, data curation, methodology, writing ‐ Original Draft, visualization, review the revised version. *Hossein Farrokhpour*: Investigation, writing ‐ Original Draft, visualization, critically revising the manuscript. *.Alireza Esteghamati*: validation, investigation, data curation, writing ‐ review & editing, review the revised version. Manouchehr Nakhjavani: conceptualization, methodology, supervision, writing ‐ review & editing, review the revised version. All authors read and approved the final revised manuscript.
